# Use of anabolic-androgenic steroids masking the diagnosis of pleural tuberculosis: a case report

**DOI:** 10.1186/1752-1947-3-30

**Published:** 2009-01-28

**Authors:** Carlos Fernández de Larrea, Aglae Duplat, Ismar Rivera-Olivero, Jacobus H de Waard

**Affiliations:** 1 Department of Internal Medicine, Hospital Vargas de Caracas, Venezuela; 2 Laboratorio de Tuberculosis, Instituto de Biomedicina, Universidad Central de Venezuela, San Nicolas a Providencia, San José, Caracas, Venezuela

## Abstract

**Introduction:**

Tuberculous pleural effusions are not always easy to diagnose but the presence of a lymphocyte-rich exudate associated with an increased adenosine deaminase level and a positive skin test result are highly sensitive diagnostic signs.

**Case presentation:**

We report a case of pleural tuberculosis in a 31-year-old white male patient from Caracas, Venezuela who was negative for human immunodeficiency virus and presented 2 weeks after injecting the anabolic-androgenic steroid nandrolone decanoate, in whom all the tests for tuberculosis were initially negative; an eosinophilic pleural effusion with a low adenosine deaminase level, a negative tuberculin skin test and negative for acid-fast bacilli staining and culture of the pleural fluid. After excluding other causes of eosinophilic pleural effusion malignant pleural effusion was suspected. The patient did not return until 4 months later. The second thoracentesis obtained a pleural fluid suggestive for tuberculosis, with a predominance of lymphocytes, an elevated adenosine deaminase level (51 U/l) and a positive tuberculin skin test. Culture of pleural fragments confirmed pleural tuberculosis.

**Conclusion:**

This case suggests that the use of an anabolic-androgenic steroid masks the definitive diagnosis of pleural tuberculosis by changing the key diagnostic parameters of the pleural fluid, a finding not previously reported. Available evidence of the effects of anabolic steroids on the immune system also suggests that patients using anabolic-androgenic steroids might be susceptible to developing tuberculosis in either reactivating a latent infection or facilitating development of the disease after a recent infection.

## Introduction

The cause of an exudative pleural effusion (EPF) is often difficult to determine, but tuberculosis (TB) must be considered, especially in countries with a high prevalence of TB. The diagnosis of a tuberculous pleural effusion is based on the Ziehl-Neelsen staining for acid-fast bacilli (AFB) and on the growth of *Mycobacterium tuberculosis *from pleural fluid or biopsy. However, if no AFB are found, cultures take 4–6 weeks to be positive, and therapeutic decisions need to be made before the results are available. A positive tuberculin skin test (TST) can be helpful, but may be negative in a third of the patients with tuberculous pleural effusions. An elevated level of the Adenosine Deaminase (ADA) activity in the pleural fluid has proven to be very sensitive and specific for the diagnosis of pleural TB, specially when the differential cell count of an exudative effusion shows a lymphocyte neutrophil ratio of 0.75 or greater [1,2]. We report a case of pleural TB in a patient who presented 2 weeks after injecting the androgenic-anabolic steroid (AAS) nandrolone decanoate, in whom all the tests for TB were initially negative. This case report reviews the possible effects of AAS therapy on the immune system and changing important diagnostic parameters in the pleural fluid.

## Case presentation

A 31-year-old white man from Caracas, Venezuela presented in the emergency room complaining of daily evening fever, night sweats, pleuritic chest pain and shortness of breath for the previous 2 weeks. Up until 2-weeks before, over 10 days, he had received a daily dose of intramuscular nandrolone (Deca-Durabolin^® ^50 mg), but was not taking any other medication. A chest X-ray showed a pleural effusion occupying 30% of the left hemi-thorax. His physical examination, blood count and serum biochemistry were otherwise unremarkable. A thoracentesis obtained clear fluid, exudative by lactate dehydrogenase (LDH) and protein levels, (310 UI/l and 4,8 gr/dl respectively) normal pH (7,46) and predominantly eosinophils (30%) in the leukocyte differential count (2500 cell per cubic milliliter) and 50% lymphocytes and 20% neutrophils. No peripheral blood eosinophilia was found. AFB staining of the pleural fluid was negative and the ADA level was normal (21 UI/L). The patient gave no history of TB contact and a TST was negative (0 mm). Culture of the pleural fluid for bacteria on blood agar and for mycobacteria on Lowenstein Jensen and Stonebrink medium was negative. A parasitic infection was suspected but serial stool examinations were negative for parasites. No definitive diagnosis was made, but malignant pleural effusion was suspected.

After thoracentesis the patient felt better and did not return to the hospital until 4-months later, when he returned to the emergency room with similar pleuritic symptoms, fever and night sweats. Additionally, he had a nonproductive cough and had lost more than 5 kilograms in weight since the previous visit. Examination of the lungs revealed decreased breath sound in the lower left hemi-thorax, but the rest of the physical examination was normal. A chest X-ray again showed the left sided pleural effusion, slightly smaller than on the previous study. A second thoracentesis obtained turbid fluid, pH 7·41, 3100 cells per cubic millimeter, 90% mononuclear and 3% eosinophils, 5,4 gr/dl proteins and 242 UI/dl LDH. Glucose, amylase levels, hematological parameters, renal and hepatic serum tests were normal. The effusion/serum ratio for proteins and LDH were 0·77 and 0·83 respectively. The ADA on the pleural fluid was elevated (51 U/L) and suggestive of tuberculosis, and the TST was positive (20 mm). Ziehl-Neelsen staining for AFB was negative both on induced sputum and pleural liquid. A pleural biopsy showed a chronic pleurisy with multiple granulomas with central necrosis, compatible with pleural TB and a culture of the pleural tissue was positive for *M. tuberculosis *after 4 weeks. The patient's symptoms disappeared after starting treatment with anti-tuberculosis drugs, and the chest X-ray showed resolution of the effusion 4 weeks later, turning completely normal.

## Discussion

Two weeks after anabolic steroid use our patient had a pleural effusion that was predominantly eosinophilic. Pleural fluid eosinophilia is very often related with conditions associated with the presence of blood or air in the pleural space [3] but our patient had no evidence of chest trauma, hemothorax, or pneumothorax. Pulmonary embolism or benign asbestos pleural effusions are other common causes of eosinophilic pleural effusion, but were also excluded, even as a parasitic or bacterial infection [3]. The relationship between symptom onset and initiation of AAS therapy in combination with the pleural fluid eosinophilia raised the suspicion of a drug-induced pleural reaction (reviewed in [4]). Certain drugs (Table 3 in [3]) can cause pleural eosinophilia. However, most of these drugs also cause peripheral eosinophilia which was not found in our patient. In addition, AAS are not listed as drugs that cause eosinophilic pleurisy. Because the frequency of malignant etiology among EPEs in general is high and has varied between 6% and 40% in different studies [3] malignancy was suspected. We did not consider a tuberculous pleural effusion as they very rarely contain high numbers of eosinophils. A prevalence of only 1·3% among 700 tuberculous pleural effusions has been reported [5]. In addition, the patient had a negative TST and pleural liquid with a normal ADA value, two important other parameters for the consideration of tuberculous pleurisy [1,2]. These three parameters however were strikingly changed at the second visit 4-months later, when the TST was positive, the pleural fluid was predominantly lymphocytic, the ADA level was elevated and tuberculous pleurisy was diagnosed.

Only a few data are available about the effects of anabolic steroids on the immune system and therefore do not allow firm conclusions but we hypothesize that the use of nandrolone could have changed the key parameters of the pleural fluid, and could have played a role in developing tuberculosis in either reactivating a latent infection or facilitating development of disease after a recent infection. It has been shown that a high dose of anabolic steroids can have significant effects on immune responses. Nandrolone decanoate directly modifies the cytokine pattern in human and murine models increasing the production of inflammatory cytokines IL-1 beta and TNF-alpha, without affecting IL-2 or IL-10 production but significantly inhibiting IFN gamma production [6] This last cytokine is essential in monocyte and macrophage Th1 activation, the most effective response against intracellular pathogens like *M. tuberculosis*. In addition, IFN gamma is a potent inductor of intracellular ADA [7] and the low level of ADA activity found in the pleural liquid just after the use of nandrolone decanoate could have been caused by the inhibitor effect of the AAS on the IFN production. This model however doesn't explain the high level of eosinophils in the pleural space just after AAS use. An eosinophilic pleural effusion is supposed to be related to IL-5 production [8], and no relation has been described between AAS and IL-5 production to explain the high level of eosinophils in the pleural space.

In conclusion, we believe that the use of AAS should be included when evaluating EPEs and should be considered a possible cause of changing pleural fluid parameters and of developing TB. It is important to stress that, as has been observed for the reactivation of TB after the use of for example glucocorticosteroids [9], the frequency of developing TB could be low in countries with a low incidence of TB (for glucocorticosteroids, 0% in the USA and Greece, 0·6% in France and 1·35% in Spain have been reported). In contrast, the frequency of development of TB after the use of glucocorticosteroids was much higher in studies performed in countries with a moderate to high incidence of TB (from 2·5% in South Korea to 13·8% in the Philippines [9]).

## Conclusion

This case suggests that patients using anabolic steroids might be susceptible to developing tuberculosis in either reactivating a latent infection or facilitating development of disease after a recent infection and that the use of nandrolone limits the diagnostic value of key parameters for the diagnosis of pleural TB, a finding not previously reported. We would like to recommend that attention should be paid to the possibility of nandrolone as a drug implicated in tuberculous eosinophilic pleurisy.

## Abbreviations

ADA: adenosine deaminase; TB: tuberculosis; AAS: anabolic-androgenic steroid; EPF: eosinophilic pleural effusion; TST: tuberculin skin test; AFB: acid-fast bacilli; LDH: lactate dehydrogenase.

## Consent

Informed written consent was obtained from the patient for publication of this manuscript.

## Competing interests

The authors declare that they have no competing interests.

## Authors' contributions

CL and AD were involved in the case directly, performed the literature search and assisted in the preparation of the manuscript. IRO was involved in the laboratory diagnosis, in the literature review and drafting of the manuscript. JW was involved in drafting the manuscript and in overall supervision. All authors read and approved the final manuscript.
